# The American Medical Association

**Published:** 1872-11-01

**Authors:** 


### Medical Examiner.

*A Semi-Monthly Journal of Medical Sciences.*

EDITED BY

N. S. DAVIS, M. D., AND F. H. DAVIS, M. D.

Chicago, November 1st, 1872.

### EDITORIAL.

### THE AMERICAN MEDICAL ASSOCIATION.

During the last five or six years, especially, a portion of the medical press in this country has indulged in a chronic habit of criticising, or, more properly, of complaining about everything done by the American Medical Association. According to this portion of the press, nothing done by the Association is done right. If a question is started, and by a proper motion is laid on the table, or its consideration indefinitely postponed without debate, it is said to have been "gagged," or "choked down." If it is discussed fairly and courteously, the discussion is "mere wrangling," and "disgraceful waste of time." If a question of ethics is examined by a committee of the oldest and ablest men in the profession, and decided by the Association, it is all represented as done by political management and intrigue. The results of the scientific work of the Association, though filling a large volume annually, are represented as worthless and discreditable. If the members indulge in liberal social entertainments, they become gormandizers and wine-bibbers; and if they abstain, they are parsimonious and unsocial. In short, the Association, in the estimation of this portion of the medical press, is an utter failure, having existed a quarter of a century, and literally accomplished nothing in the way of promoting either the scientific, educational, or moral interests of the profession. As a rule (and we can at present think of no exceptions), this croaking is done through the columns of those papers or journals whose editors have either never attended or actively participated in the meetings of the Association. In one or two instances they may have sent a reporter to note the proceedings; but, for the most part, they have relied on the imperfect reports of the secular press. Hitherto we have paid little attention to these fault-finders, thinking that they would either get weary of their own work, or commit themselves to some definite plans for remedying the evils of which they complain. And during the present year we have been gratified to find in the columns of most of them positive suggestions for improvement. The *Medical Record*, of New York, for July 1st, took the lead; and like a truly scientific physician, the editor first points out the *cause* of the existing evils. His startling discovery in this respect, together, with his proposed remedies, we give in his own words, as follows:

To Dr. Davis, more than to any one man, more indeed than to any number of men, the Association is indebted for whatever of evil or of good it has accomplished. A natural and practiced speaker, with a talent and taste for business management that is quite rare among physicians, he has from the foundation of the association been one of its leading spirits, and now almost entirely controls it. A large proportion of the delegates are utterly ignorant of the rules of the Association, and are utterly indifferent to its business management; while Dr. Davis is exhaustively familiar with every fact of the Association from its birth until now, and is thoroughly enthusiastic for its welfare; has, indeed, made this matter a specialty of his life. The delegates who vote at all find it easier to vote with Dr. Davis, whom they suppose to know more than any other man about any subject that is presented, than to study it up for themselves, and hence it happens that we have the strange spectacle of a thousand men—the best representatives of the medical profession in America—yielding to one-man power with the most absolute satisfaction and unconcern. If Dr. Davis had gone *alone* into the hall at Philadelphia, made himself chairman, secretary, and treasurer of the Association; if he had proposed and seconded and voted on all the resolutions, the final result would have been just about as it was with a thousand men around him. It was on the Friday morning session that the relation of Dr. Davis to the Association appeared most conspicuous. The order of business was the disposal of the business on the secretary's table. The secretary began to read the resolutions that had been handed him, and to make motions concerning the publication or non-publication of papers. Then there followed an exhibition, the like of which eye hath not seen nor ear heard, neither hath it entered into the heart of man to conceive—an exhibition which probably is without a parallel in the history of public conventions. Dr. Davis, who had taken a seat near the pulpit, eagerly watched every resolution as it was read, and every motion that was made, and successively disposed of them according to his own wish. Other members made motions to table, to reconsider, or to do this or that; but nothing was finally settled contrary to Davis' wishes. Other voices were heard from near and from far in feeble protest, but all were drowned in this one voice. Meanwhile, from every part of the church, from the pulpit and from the vestibule, men were seen rushing to Davis, and in tones of humility and reverence, such as are supposed to be appropriate for those who address the Almighty—were craving his permission to make or to modify resolutions, to insert this or take out that, as though all well knew that it would be useless to attempt to carry any measure to which he was opposed. The king received his subjects with mingled firmness and gentleness, as every good ruler should; some were favorably answered, others doubtfully, and others sent empty away, sad and discouraged. We give one illustration out of many that might be given. When Dr. Reese's carefully-worded resolution in regard to woman was brought up, a few prominent members seemed to be disturbed lest the Association might again disgrace itself and the country on this subject, and there appeared to be quite a general feeling that perhaps it might not be impossible to redeem the bad record of the past quarter of a century. Then arose Dr. Davis, and half jestingly said that he "preferred to talk with the ladies in Fairmount Park, than talk about them here;" and at once, in the twinkling of an eye, the representatives of forty thousand educated physicians said, Amen!

In short, Dr. Davis was the Association. It was a strange, a sublime, a picturesque sight—this voluntary, unanimous, unswerving and unconscious yielding of a thousand wills to one. If there was any single measure of the Association at the meeting that was at all displeasing to Dr. Davis, he has only himself to blame; he had only to breathe a wish, and it was his.

These words are not in censure of Dr. Davis; they are rather in his praise. But for his watchfulness, care, and supreme influence, the Association might have passed we know not how many absurd resolutions.

Now, it would be very difficult for any one man to fully represent the views of the leading minds of the profession in this country on all the great questions that arise; and Dr. Davis is certainly not the man, for on the woman question, and on certain matters of ethics, he no more represents the best physicians of America than Dr. Martyn Payne's work on Materialism represents modern science. He has a perfect right to his views. He has as much right to worship the eighteenth century as we have to worship the nineteenth. If a man takes pride in being a quarter of a century behind the age, there is no law to interfere with him. He has a legal, though scarcely a scientific or moral right to Bourbonize the American Medical Association; and if the members of the Association allow themselves to vote for wicked and cruel and iniquitous measures, that would disgrace the lowest order of politicians, they must bear at least a part of the blame.

It is not our purpose here to find fault, but to explain what to many has been a great mystery, the failure of the American Medical Association to represent the American Medical profession.

We close with these suggestions: First, let the meetings of the Association be held with closed doors. What would be thought if all the petty squabbles of our county societies were regularly published in the newspapers, so that the world could know by heart every blunder that we make? Why should the vaster, more important, and annually recurring blunders of the American Medical Association be made the property of the laity, and advertised over the land to our perpetual shame? Our friend President Yandell, in his closing address, said that the session "had inspired the spectators in the gallery with a higher respect for the profession in America." Now we venture to say that if the few straggling spectators in the gallery had brains enough to be inspired with anything, it was with a *lower* respect for the medical profession in America; and their feelings must have been shared by all intelligent readers of newspapers.*

*A few years ago, when the Association met in New Haven, a resident of that city, a calm, wise, and judicious man, who had no prejudice against science or our profession, a man in no way cynical, but thoroughly genial as well as scholarly, and who had heard and read the debates in the morning sessions, made this remark: "*The American Medical Association is the greatest of all humbugs.*"

Secondly, as far as possible re-elect delegates so that the Association shall comprise men who know something of its history, rules and management.

Thirdly, banish to everlasting darkness that mass of useless lumber called the Transactions, and all discussions about them, and let the papers that are worth anything be published, in the same century in which they were written, and where they will be read by somebody besides the Permanent Secretary.

Fourthly, let all the important work of the Association be done by *large committees, fairly chosen.*

A body of a thousand men cannot determine complicated questions in a breath; there is a need of the study, and care, and reading, and conference, and time, that only a committee can give.

The charge has been made that the committee on ethics, that has made so much trouble in the country and has done so much dishonor to our profession and our country before the world, is a *packed* committee. We have no evidence of the truth of this charge; but hereafter let the committees be chosen in such a way that no similar charge can be entertained.

Let these suggestions be carried out, and the American Medical Association may become a power instead of a farce in the profession. Its suggestions will be received with respect, even when they are not adopted, and it will elevate instead of degrading the science of America.

So it appears, by the *Record*, that the senior editor of this journal is the all-controlling spirit, who must answer for all the sins of the Association.

We will not stop, however, to waste a moment on personalities. We have given the quotation as a fair specimen of the way nearly all these fault-finders draw on their imaginations for their facts. We are told, in substance, that the *thousand* members assembled from all parts of the country needed only to know the wishes of Dr. Davis on any subject, and they were at once complied with. And the doings of the morning session of Friday are specified as an example, and a vivid picture is drawn of what one might imagine the writer saw passing before him on that occasion. Being wholly unconscious of any such scene as represented in the *Record*, we have taken the trouble to refer to the official record of the proceedings of Friday, and find that during the general session of that day there were thirty-four subjects presented and acted on by the Association. Four of these were resolutions relating to eminent deceased members; eight, resolutions of thanks to different parties; and five were simple motions to refer reports of sections and committees to the committee of publication. Of course, no one thought of voting against any of these. The remaining seventeen related to items of miscellaneous business; and, strange as it may seem after reading the above quotation from the *Medical Record*, no less than eight of these were *not* disposed of in accordance with either the votes or wishes of Dr. N. S. Davis. It appears that he made only two motions during that session: one to refer a certain matter to a special committee, and the other to adjourn. He occupied the floor three times: once in reply to the sweeping assertion of Dr. Gross, that in regard to medical education the Association had been an "utter failure;" once in regard to the proper method of disposing of papers and reports in the Sections; and the third time was the playful remark that he would rather meet the ladies socially in the Park (where the Association had been invited to spend the afternoon), than to hear their merits discussed in that assembly. So far from opposing the "carefully-worded" resolution of Dr. Reese, in relation to the medical education of woman, on the first motion, by which it was laid on the table, he did not vote. At a subsequent stage of the meeting, when Drs. Rogers and Busey explained that the resolution had not probably been understood, and asked for a reconsideration, he voted promptly in favor of taking it from the table and giving it a rehearing.

The truth was, he regarded the resolution as of no practical importance, and felt entirely indifferent as to how it might be disposed of, provided its movers had a fair hearing. And it was not until it became evident, on its second consideration, that the first clause would elicit another discussion on "the rights and capabilities of woman," that he rose and reminded the members that it was within a few minutes of the proper time for adjournment, and expressed his preference for the society of the ladies in the Park, over the hearing of a discussion on their rights and wrongs on that floor. And if the assembled *thousand* said amen to the suggestion, it was not so much from any magic influence of one man, as it was because the suggestion was in accordance with common sense. It is evident from the whole tenor of the article in the *Medical Record*, that it is the supposed position of Dr. Davis on the "woman question," and on the committee on ethics, that constitutes him a Bourbon and a representative of the eighteenth instead of the nineteenth century.

But here, too, our friend of the *Record* has drawn on his imagination, instead of the facts of history. If he had taken pains to refer to the record of proceedings of the meeting of the Association in Washington, 1870, he would have found that the committee on ethics, of which Dr. Davis was a member, reported unanimously in favor of admitting the delegates from the Woman's College and the Woman's Hospital, of Philadelphia, there being nothing in the Constitution or the By-laws to prevent it. But the Association refused to adopt the report of the committee.

The following year, at San Francisco, he was again a member of the committee on ethics, to whom was referred the credentials of the delegate from the Woman's Hospital of Philadelphia, and said committee declined to express any opinion on the subject until the Association had acted on an amendment to the Constitution, proposed by Dr. Henry Hartshorne, touching the same subject, and the adoption or rejection of which would decide the question referred to the committee. And at the last meeting in Philadelphia, the committee on ethics had no question referred to it touching the rights of woman or woman's institutions. And as to the other so-called great question of the age, the rights and social status of the colored men, it has never been referred to, or considered by, the committee on ethics, either directly or indirectly. Neither has the committee ever made any report touching that subject. There is not in the Constitution, By-Laws, Ordinances, and Code of Ethics of the American Medical Association, one word or syllable in relation to distinctions of sex, race, or color. And as an individual member of the Association, Dr. Davis has opposed all propositions to change this position in any of these respects, believing it better that every annual meeting should be left free to exercise its own judgment, untrammeled by constitutional barriers. Whenever his opinions or council have been asked touching these matters, he has uniformly advised that whenever a delegate presents properly attested credentials from any society or institution *entitled to representation* in the Association, such delegate should be admitted without a single question in regard to individual sex, race, or color. And if a delegate presented credentials from a society or institution concerning which the registration committee had charges or information of ineligibility to representation, such committee should report the facts to the Association for its action.

Having thus disposed of the more personal aspect of this subject, we shall in future numbers devote a few pages to a careful examination of the various propositions for improving the organization and working power of the Association, hoping that it may prove both interesting and useful to our readers.

Large Doses of Opium.—We publish the article of Dr. Carpenter, in the present number of the Examiner, because we think it desirable that such facts, when they exist, should be put on record for reference; yet we cannot recommend the use of such enormous quantities of opium. To give from three to five grains of opium in substance, every fifteen, thirty, or even sixty minutes, is faster than the mucous membrane of the stomach will absord it; and there must always be great danger of such an accumulation of the drug as will endanger the life of the patient, whenever the intensity of the pain abates. If we admit Dr. C. to be correct in the supposition that the opium he used was of good quality, we cannot explain the results, except on the assumption that the stomach was in such a condition it did not absorb the active constituents of the drug, and they passed the bowels with the fœces.

---

IMPOSITION.—We have received a *hand-bill* from Momenee, Ill., headed "To the Sick," in which Drs. Gray and Cole make large promises in regard to curing all chronic diseases; and as references, at the bottom of the bill, are the names of "N. S. Davis, M. D., LL.D.; Moses M. Gunn, M.D.; and S. Small, M.D., of Chicago." Having never authorized any such use of our name, either with or without the anomalous "LL.D.," the only recommendation we can give concerning Drs. Gray and Cole, is, that they be rode on a rail out of every town in which they post such a hand-bill.

